# Toxicity, repellency and flushing out in *Triatoma infestans* (Hemiptera: Reduviidae) exposed to the repellents DEET and IR3535

**DOI:** 10.7717/peerj.3292

**Published:** 2017-05-18

**Authors:** Mercedes M.N. Reynoso, Emilia A. Seccacini, Javier A. Calcagno, Eduardo N. Zerba, Raúl A. Alzogaray

**Affiliations:** 1 UNIDEF, CITEDEF, CONICET, CIPEIN, Villa Martelli, Buenos Aires, Argentina; 2 Centro de Estudios Biomédicos, Biotecnológicos, Ambientales y de Diagnóstico (CEBBAD), Departamento de Ciencias Naturales y Antropológicas, CONICET, Ciudad Autónoma de Buenos Aires, Argentina; 3 Instituto de Investigación e Ingeniería Ambiental (3IA), Universidad Nacional de San Martín (UNSAM), San Martín, Buenos Aires, Argentina

**Keywords:** Diethyltoluamide, Ethyl 3-[acetyl(butyl)amino]propanoate, Blood-sucking bugs, Locomotor activity

## Abstract

DEET and IR3535 are insect repellents present worldwide in commercial products; their efficacy has been mainly evaluated in mosquitoes. This study compares the toxicological effects and the behavioral responses induced by both repellents on the blood-sucking bug *Triatoma infestans* Klug (Hemiptera: Reduviidae), one of the main vectors of Chagas disease. When applied topically, the Median Lethal Dose (72 h) for DEET was 220.8 µg/insect. Using IR3535, topical application of 500 µg/insect killed no nymphs. The minimum concentration that produced repellency was the same for both compounds: 1,15 µg/cm^2^. The effect of a mixture DEET:IR3535 1:1 was similar to that of their pure components. Flushing out was assessed in a chamber with a shelter containing groups of ten nymphs. The repellents were aerosolized on the shelter and the number of insects leaving it was recorded for 60 min. During that time, 0.006 g/m^3^ of the positive control tetramethrin flushed out 76.7% of the nymphs, while 1.76 g/m^3^ of DEET or IR3535 flushed out 30 and 0%, respectively. The concentrations required for both compounds to produce toxicity or flushing out are too high to have any practical applications. However, they showed a promising repellency. Additional research should be done to evaluate their possible use for personal protection against *T. infestans* bites.

## Introduction

An insect repellent has been defined as “something that causes insects to make oriented movements away from its source” (White & Moore, 2015). The main use for these substances is personal protection against the bite of hematophagous insects (Debboun & Strickman, 2013). There is a worldwide market of repellent products that contain different active ingredients (Xue, Muller & Day, 2015). Most contain N,N-diethyl-3-methylbenzamide (DEET), an active ingredient that has been used for over 60 years all over the world (White & Moore, 2015). DEET is a highly efficient repellent for a broad spectrum of insect species; furthermore, its toxicity in mammals is very slow (EPA, 2000). The United States Environmental Protection Agency (EPA) considers that DEET does not present any risk of concern to human health (Antwi, Shama & Peterson, 2008). However, it can occasionally cause skin reactions and convulsions, especially in children (Osimitz et al., 2010).

The insect repellent ethyl 3-[acetyl(butyl)amino]propanoate (IR3535) was developed by Merck & Co. in 1975. Compared to DEET, oral or dermal exposure to IR3535 is less toxic and less irritant to mammals (WHO, 2001a; Nentwig, 2003). After more than 30 years of use, the only adverse effect reported for IR3535 is irritation to eyes (Puccetti, 2007). According to the WHO, it is “effective and safe for use in human beings” (WHO, 2001b). In Argentina, DEET and IR3535 are authorized as components in several repellent products (ANMAT, 2012).

Chagas disease, the most severe parasitic disease of the American continent, is caused by the protozoan *Trypanosoma cruzi* (Chagas, 1909) (Lent & Wygodzinsky, 1979). *T. cruzi* is transmitted to humans and other vertebrates by domestic, peridomestic, or sylvatic insects of the Triatominae family (Stevens et al., 2011). The triatomine *Triatoma infestans* (Klug, 1834) is the most important vector of *T. cruzi* in Argentina, Bolivia, Paraguay and Perú (Schofield and Gorla, 2010). In Latin America, Chagas disease affects more than 5.740.000 people (World Health Organization (WHO), 2015).

Different studies have assessed the behavioral response of Chagas disease vectors to synthetic and natural repellents under laboratory conditions (Alzogaray, Fontán & Zerba, 2000; Coelho, De Paula & Spíndola, 2006; Ferrero, González & Chopa, 2006; Abramson, Aldana & Sulbaran, 2007; Mello et al., 2007; Sfara, Zerba & Alzogaray, 2009; López et al., 2011; Pohlit et al., 2011; Avelar-Freitas et al., 2012; Gomes & Favero, 2013). The effects of DEET were barely explored in *T. infestans*, and there are no reports on the effects of IR3535 on triatomines. On the other hand, there is evidence that both repellents have insecticidal activity in house fly and mosquitoes (Pridgeon, Bernier & Becnel, 2009; Swale et al., 2014). Based on this background, the objective of this work was to compare the toxicological and behavioral responses of fifth-instar nymphs of *T. infestans* when exposed to DEET or IR3535.

## Materials and Methods

### Biological material

Fifth-instar nymphs of *T. infestans*, 7–15 days old after last ecdysis, were provided from a laboratory colony maintained by the Centro de Referencia de Vectores (Santa María de Punilla, Córdoba, Argentina). They were kept at 26 ± 2 °C and 60–90% RH until each experiment.

### Chemicals

DEET (97%) was purchased from Sigma Aldrich (Buenos Aires, Argentina), IR3535 (99.6%) was a gift from Merck Argentina (Buenos Aires, Argentina), and analytical grade acetone was acquired from Merck Germany (Darmstadt, Germany).

### Toxicity bioassays

Six groups of ten nymphs were separated and each was randomly assigned to one of the following treatments: acetone alone (negative control), 31.25, 62.5, 125, 250 or 500 µg of DEET per insect. These concentrations were chosen after a preliminary assay. Treatment were applied using a microsyringe with a dispenser (Hamilton, Reno, NE), and each nymph received 1 µl solution on the abdomen. A similar bioassay was not performed with IR3535 because 500 µg/insect of this repellent produced no mortality.

Immediately after the treatment, nymphs were placed in a plastic container (10 cm high, 8 cm in diameter) closed with a gauze held with a rubber band. The container was maintained in an incubator FOC 225E provided with a thermoregulation system (Velp Scientifica, Usmate, Italy) programmed at 26 ± 2 °C and 60–90% RH. The number of affected nymphs was recorded 72 h after the treatment.

To quantify the toxicity of DEET and IR3535, a circle of filter paper 15 cm in diameter (101 FAST, Hangzhou Xinxing Paper Industry and Co., Ltd., Fuyang, China) was placed in a plastic container (32 cm long, 25 cm wide, and 8 cm high; Colombraro, Buenos Aires, Argentina). The treated nymphs were then carefully dropped in the centre of the paper circle and observed for 1 min. According to preliminary observations, control nymphs abandon the paper circle in less than 5 s, following an approximately straight line toward the side of the plastic container. After these observations, a nymph remaining for at least 1 min on the paper circle and showing symptoms of intoxication (difficulty walking or no movement after being gently touched with a soft tweezer) was considered dead. Three independent replicates were made for each assay, and the Median Lethal Dose (*LD50*) was calculated.

### Recording equipment

A black and white closed-circuit video camera (VC 1910, Sanyo Electrical Co., Tokyo, Japan) and an image analyser (Videomex V, Columbus Instruments, Columbus, OH) were used to evaluate repellency. The video camera records the movement of the nymph placed in the experimental arena and sends an analogical signal to the image analyser, where it is digitalized. Thus, the nymph appears as a white silhouette (pixels “on”) on the image analyser screen, while the filter paper appears as a black surface (pixels “off”). The Multiple Zone Motion Monitor software compares consecutive frames captured by the camera and records the number of pixels that change from “on” to “off” or vice versa. This software calculates two parameters: (a) Motion (M), the sum of pixels that changed during the assay, and (b) Area (A), the number of pixels that remained “on” (it represents the average area occupied by the nymph).

### Repellency bioassays

As a first approach to quantify comparatively the repellence of these compounds, we used a preference test such as is commonly used to evaluate repellent effects on walking insects (Scheffler & Dombrowski, 1992; Aggarwal et al., 2001). A circle of filter paper 110 mm in diameter (101 FAST, Hangzhou Xinxing Paper Industry and Co., Ltd., Fuyang, China) was cut into halves. One half was treated with 0.25 ml of DEET or IR3535 dissolved in acetone, and the other half was treated with 0.25 ml of acetone alone. After the solvent evaporated, both halves were stuck back together with adhesive tape on the underside, and the circle was placed on a horizontal surface. A glass ring (2.5 cm high, 10 cm in diameter) was used to prevent the insects left the experimental arena. Finally, a nymph was gently deposited on the centre of the arena.

Each nymph was randomly assigned to one of six treatments: solvent alone (control), 0.38, 1.15, 3.43, 10.33, or 31.00 µg/cm^2^ of each repellent alone. A mixture of DEET:IR3535 1:1 was also tested at the same concentrations.

The image analyser recorded the nymph movement on each zone of the filter paper for 15 min. Results were used to calculate a Distribution Coefficient (DC) (Moretti, Zerba & Alzogaray, 2013):

\begin{eqnarray*}DC=(AT-At)/AT. \end{eqnarray*}

*AT* is the area occupied by the nymph throughout the assay, *At* is the area occupied by the nymph in the treated zone of the experimental arena. Values of *DC* vary between 0 (maximum attraction to the treated zone) and 1 (maximum repellence). Values close to 0.5 indicate that the insect spent approximately the same amount of time on each zone.

The experimental arena was illuminated with a cold light lamp (22 watts; Luxa, Shangai, China) located at the zenith. Temperature varied between 24 and 28 °C. Each assay was repeated four times. Replicates were carried out on different days with newly prepared solutions, and each insect was used only once.

### Flushing out bioassays

**Figure 1:**
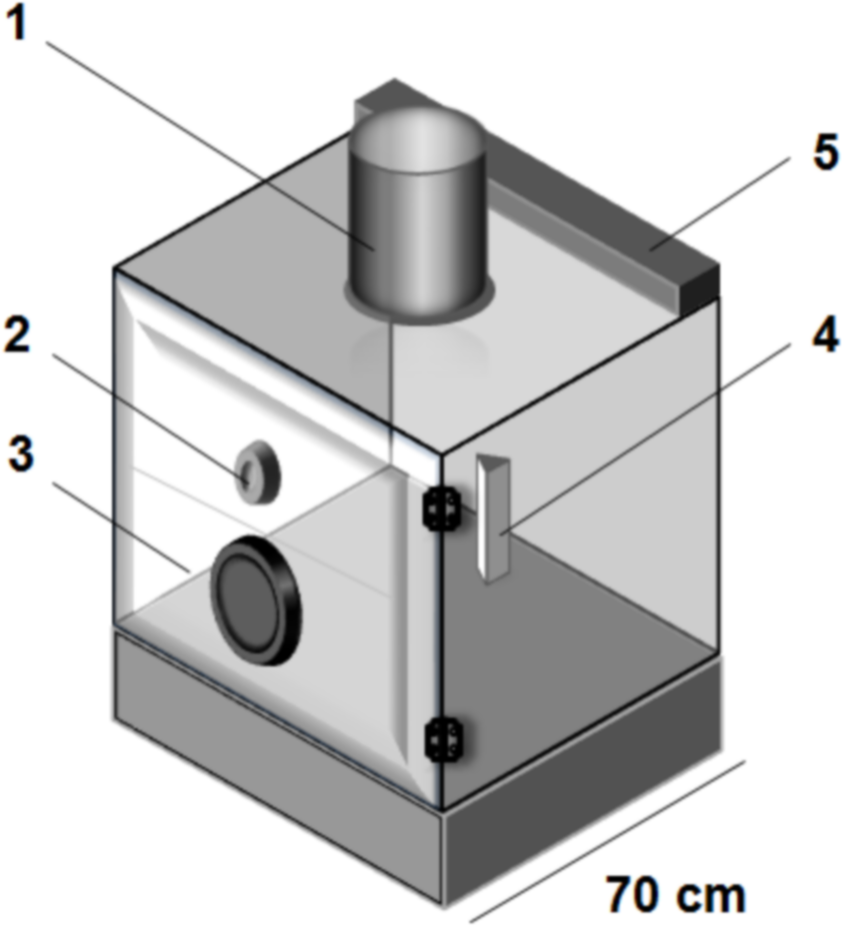
Glass chamber used for flushing out bioassays. 1, Exhaust fan (it is used to exhaust the contaminated air out of the glass chamber after each assay); 2, hole sealed with a rubber stopper; 3, front panel; 4, black cardboard triangular hollow prism (insects refuge); 5, two cold light tubes.

Bioassays were performed inside a glass chamber (70 × 70 × 70 cm), illuminated by two cold light tubes of 20 watts each (Osram, Buenos Aires, Argentina) placed externally at the upper rear corner (Fig. 1). The front panel had a hole (5 cm in diameter) through which the flushing out agents were aerosolized into the chamber. Room temperature was maintained at 26 ± 2 °C. A black cardboard shaped as a triangular hollow prism (3 cm × 15 cm high), with its two ends opened, was located vertically inside the glass chamber (5 cm from the back wall and equidistant from the lateral walls). Ten fifth instar nymphs were gently released inside the black cardboard refuge and were allowed 15 min of familiarization. Following this, 1 ml of repellent in acetone (150, 300 or 600 mg/ml, equivalent to 0.44, 0.88, and 1.76 g/m^3^, respectively) was aerosolized through the front hole of the chamber using a glass sprayer. Compressed nitrogen was used as the carrier (3.5–3.8 psi). After the treatment, the hole in the chamber was sealed with a rubber stopper, and the number of insects leaving the refuge was recorded every 5 min during 1 h. One ml of acetone alone was aerosolized as a negative control and a solution of tetramethrin (0.002 mg/ml, equivalent to 0.006 g/m^3^) was applied as a positive control. Three independent replicates were performed for each treatment.

### Statistical analysis

*LD50* values with their respective 95% Confidence Limits were calculated using the PoloPlus 2.0 programme (LeOra Software, 2002). Results from the repellency flushing out bioassays were analysed using one-way ANOVA, followed by Tukey’s *post hoc* comparisons when *P* < 0.05. Results of repellency were also used to calculate linear regressions.

## Results

Topical application of DEET and IR3535 showed very low toxicity on fifth instar nymphs of *T. infestans* (Table 1). The *LD50* at 72 h for DEET was 220.8 µg/insect. No mortality was observed after topical application of 500 µg/insect of IR3535.

**Table 1:** Toxicity of DEET on fifth instar nymphs of *Triatoma infestans*.

	LD50^a^ (µg/insect) (95% CL)^b^	*N*	Slope ± SE	Chi-square
DEET	220.8 (167.8–313.0)	180	1.9 ± 0.3	1.99
IR3535	>500.0^c^	–	–	–

**Notes.**

a Median Lethal Dose at 72 h.

b 95% Confidence Limit.

c No mortality was observed when this dose was applied.

**Figure 2:**
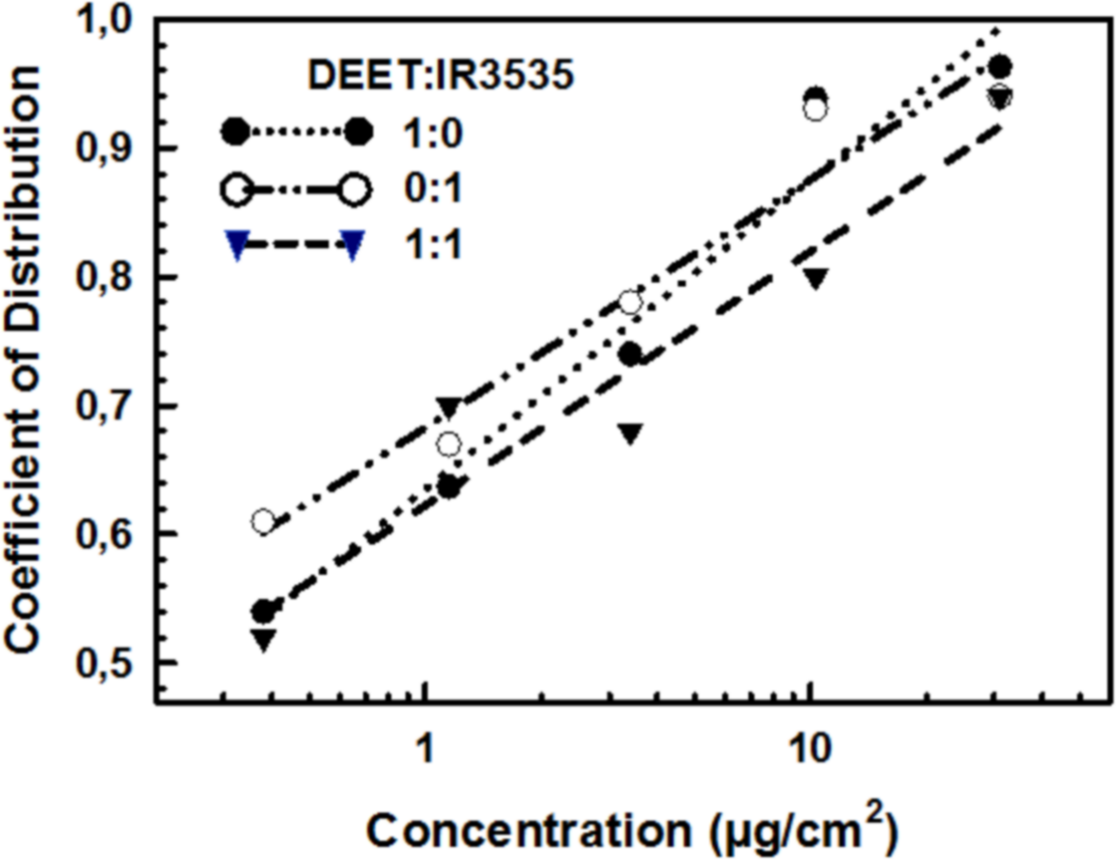
Repellency of pure and mixed DEET and IR3535 in fifth instar nymphs of *Triatoma infestans*. Distribution Coefficient = (*AT* − *At*)∕*AT*, where *AT* is the total area occupied by the nymph on the arena, and *At* is the area occupied by the nymph on the treated zone throughout the experiment.

The repellent effect of pure and mixed solutions of DEET and IR3535, applied at concentrations ranging between 0.38 and 31.00 µg/cm^2^, increased as a linear function of log concentration (Fig. 2 and Table 2). The values of *r*^2^ varied between 0.922 and 0.963, indicating a good fit to the model in all cases (Table 2). The minimum concentration that was significantly different from control (i.e., the minimum concentration that produced repellency) was the same for both substances: 1.15 µg/cm^2^ (*p* < 0.05). The effect of the mixture 1:1 was similar to the effects of their separate components (Table 3). In other words, neither synergy nor antagonism was observed.

**Table 2:** Linear regressions for the independent and joint repellency of DEET and IR3535 on fifth instar nymphs of *Triatoma infestans*.

DEET:IR3535	Regression	*r*^2^	*F*	*df*	*P*
1:0	*DC* = 0.635 + 0.242 logC	0.963	77.920	1, 3	0.003
0:1	*DC* = 0.683 + 0.193 logC	0.951	57.779	1, 3	0.005
1:1	*DC* = 0.622 + 0.199 logC	0.922	35.613	1, 3	0.009

**Notes.**

*DC*, Distribution Coefficient = (*AT* − *At*)∕*AT*, where *AT* is the total area occupied by the nymph on the arena, and *At* is the area occupied by nymphs on the treated zone throughout the experiment; logC, log of concentration.

**Figure 3:**
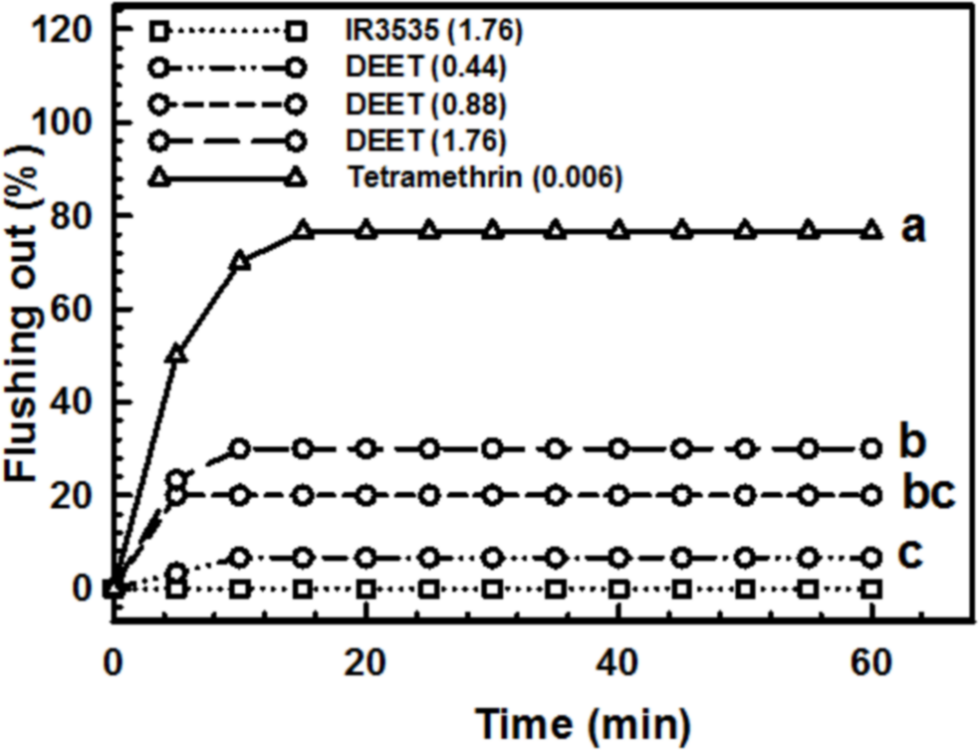
Flushing out in fifth instar nymphs of *Triatoma infestans* exposed to aerosolized DEET. In the legend, values in brackets are expressed in units of g/m^3^. Different letters indicate significant differences (*P* < 0.05) (IR3535 produced 0% of flushing out in all replicates, so it was not included in the ANOVA). All replicates of IR3535 produced 0% of flushing out.

No flushed out nymphs were observed when acetone or IR3535 alone were aerosolized on the insect refuges. Tetramethrin and DEET produced a significant flushing out (*F* = 26.51; *df* = 3, 8; *p* < 0.001) (Fig. 3). The positive control tetramethrin flushed out 76.7% of nymphs when applied at 0.006 g/m^3^. Flushing out by DEET increased as the concentration increased. However, despite the high concentrations of this repellent used (0.44–1.76 g/m^3^), none of them exceeded 40% of flushing out during the experimental time. IR3535 did not flushed out any nymph even at 1.76 g/m^3^.

**Table 3:** Statistical analysis of different concentrations of pure or mixed DEET and IR3535.

Concentration (µg/cm^2^)	DC^a^(SE)			*F*	*df*	*P*
	DEET:IR3535					
	1:0	0:1	1:1			
0.38	0.54a (0.06)	0.61a (0.03)	0.52a (0.04)	0.840	2, 9	0.463
1.15	0.64a (0.06)	0.67a (0.07)	0.70ab (0.06)	0.198	2, 9	0.824
3.4	0.74ab (0.06)	0.78ab (0.07)	0.68ab (0.04)	0.754	2, 9	0.498
10.3	0.94bc (0.03)	0.93b (0.03)	0.80bc (0.06)	3.271	2, 9	0.086
31	0.96c (0.02)	0.94b (0.03)	0.92c (0.03)	0.274	2, 9	0.767
*F*	24.0	16.1	11.7			
*df*	5, 18	5, 18	5, 18			
*P*	<0.001	<0.001	<0.001			

**Notes.**

a Distribution Coefficient = (*AT* − *At*)∕*AT*, where *AT* is the total area occupied by the nymph on the arena, and *At* is the area occupied by nymphs on the treated zone throughout the experiment. In each column, values followed by the same lowercase letter are not significantly different (*P* > 0.05). Statistical parameters are from one-way ANOVA.

## Discussion

In this work, the following was studied for the first time in *T. infestans*: (a) DEET and IR3535 toxicity, (b) the behavioral response to IR3535 alone or mixed with DEET, and (c) the flushing out effect of both compounds.

There are very few studies on the insecticidal properties of DEET and IR3535. In the house fly and the mosquitoes *Aedes aegypti* (Linnaeus, 1782) and *Anopheles gambiae* (Giles, 1902), DEET showed moderate toxicity when a few micrograms were applied topically (Swale et al., 2014). It was also slightly more toxic than IR3535 in several mosquito species (Pridgeon, Bernier & Becnel, 2009).

Here, the LD50 (72 h) for DEET was 220.8 µg/insect. This is an extremely low toxicity when compared, for example, to deltamethrin, a pyrethroid widely used for controlling *T. infestans*. The Median Lethal Dose (72 h) of this insecticide in fifth instar nymphs is 1.27 ng/insect (De Oliveira Filho, 1999). Toxicity of DEET in triatomines was even lower when applied as films on filter paper on *T. rubida* (Uhler, 1894) (Terriquez et al., 2013). On the other hand, the topical application of a high concentration of IR3535 produced no toxic effects on the nymphs. The same result was reported in *R. prolixus* (Alzogaray, 2016).

The primary site of action by which this repellent exerts its insecticidal activity has not yet been identified. DEET inhibits house fly and mosquito acetylcholinesterase activity, but only at very high concentrations (Corbel et al., 2009). Neurophysiological studies suggest that the octopaminergic receptor of insects might be its target (Swale et al., 2014).

Several reasons related to toxicokinetic and toxicodynamic processes could be the cause of the low toxicity of DEET and IR3535 in triatomines. For example, a low rate of cuticular penetration or a high rate of biotransformation might explain this characteristic. In *R. prolixus*, mixed function microsomal oxidases could be involved in the biotransformation of DEET because when these enzymes are inhibited with pyperonil butoxide, the toxicity of DEET is doubled (Alzogaray, 2016).

The repellent activity of these two compounds have been mainly studied in mosquitoes. IR3535 appeared to be as efficient as DEET in *Aedes* and *Culex* spp., but less efficient in *Anopheles* (Barnard et al., 2002; Fradin & Day, 2002; Barnard & Xue, 2004; Cilek, Petersen & Hallmon, 2004; N’Guessan et al., 2006). In the present work, DEET and IR3535 were equally repellent to fifth instar nymphs of *T. infestans*, presenting the same minimum concentration that produced repellency (1.15 µg/cm^2^).

Pyrethroids and some botanical monoterpenes induce a non-directional increase in the locomotor activity of insects (Gammon, 1978; Alzogaray, Fontán & Zerba, 1997; Moretti, Zerba & Alzogaray, 2013). If the exposed insects are hidden in a shelter, they leave it by chance. This phenomenon is called flushing-out and is exploited to detect the presence of triatomines (Pinchin, De Oliveira Filho & Pereira, 1980). In Argentinian rural areas where Chagas disease is endemic, sanitary agents use aerosolized tetramethrin to flush out *T. infestans* from their shelters (Gürtler et al., 1993). Flushing out allows determining whether a domicile is infested with triatomines; it is also used to evaluate the efficacy of an insecticide treatment and study the reinfestation of treated houses (Gürtler et al., 2001). In previous years, resistance to pyrethroids has been reported in *T. infestans* populations from Argentina and Bolivia (Picollo et al., 2005; Roca-Acevedo, Picollo & Santo-Orihuela, 2013). The individuals from these populations are resistant to both knock down and hyperactivation produced by pyrethroids (Sfara, Zerba & Alzogaray, 2006). It is therefore highly important to identify alternative flushing out agents. Among natural compounds, isobutyric acid, 3-pentanol, 3-methyl-1-butanol (Minoli et al., 2013), and several monoterpenes (Moretti, Zerba & Alzogaray, 2015) showed flushing out activity on triatomines.

In the present work, DEET showed a very weak flushing out capacity compared to tetramethrin, a pyrethroid usually used by sanitary agents in Argentina to flush out triatomines. IR3535 produced no flushing out at all. Considering that hyperactivity is a symptom of intoxication, these results could be considered a consequence of the very low toxicity of these compounds in *T. infestans*.

Regrettably, the concentrations of DEET and IR3535 required to produce toxicity or flushing out in these species seem too high to have any practical applications. However, both compounds showed a similar and promisory repellency. Additional research should be done to evaluate the possible use of these compounds for personal protection against *T. infestans* bites. In particular, it may be worth to look for synergistic interactions with other compounds; for example, the botanical monoterpene eucalyptol, which showed repellent and insecticidal activity in *T. infestans* (Moretti, Zerba & Alzogaray, 2013).

##  Supplemental Information

10.7717/peerj.3292/supp-1 Data S1 Records of toxicity and behaviour modification of Triatoma infestans exposed to the repellents DEET and IR3535

## Additional Information and Declarations

### Data Availability

The following information was supplied regarding data availability: The raw data has been supplied as a Data S1.
