# Supplementary material for: Toxicity, repellency and flushing out in Triatoma infestans (Hemiptera: Reduviidae) exposed to the repellents DEET and IR3535
Source: PeerJ. 2017 May 18;5:e3292. doi: 10.7717/peerj.3292 (PMC5438576; doi:10.7717/peerj.3292)
Supplement: Data S1 [file peerj-05-3292-s001.docx]

**Toxicity, repellency and flushing out in *Triatoma infestans* (Hemiptera: Reduviidae) exposed to the repellents DEET and IR3535**

**Raw data**

Table 1 (Toxicity of DEET on fifth instar nymphs of *T. infestans*)

| Concentration  (µg/insect) | control | | |
| --- | --- | --- | --- |
|  | R1 | R2 | R3 |
| 0 (control) | 0 | 0 | 0 |
| 31.25 | 0 | 0 | 1 |
| 62.5 | 1 | 1 | 1 |
| 125 | 3 | 3 | 3 |
| 250 | 8 | 3 | 4 |
| 500 | 7 | 7 | 10 |

Fig 2 (Repellency of pure and mixed DEET and IR3535 in fifth instar nymphs of *T. infestans*) and Tables 2 (Linear regressions for the independent and joint repellency of DEET and IR3535 on fifth instar nymphs of *T. infestans*) and 3 (Statistical analysis of different concentrations of pure or mixed DEET and IR3535).

| Concentration (µg/cm²) | DEET | | | | IR3535 | | | | DEET:IR (1:1) | | | |
| --- | --- | --- | --- | --- | --- | --- | --- | --- | --- | --- | --- | --- |
|  | R1 | R2 | R3 | R4 | R1 | R2 | R3 | R4 | R1 | R1 | R2 | R3 |
| 0.38 | 0.52 | 0.66 | 0.63 | 0.61 | 0.48 | 0.42 | 0.55 | 0.71 | 0.34 | 0.5 | 0.61 | 0.41 |
| 1.15 | 0.76 | 0.77 | 0.69 | 0.44 | 0.59 | 0.69 | 0.76 | 0.51 | 0.63 | 0.7 | 0.59 | 0.63 |
| 3.50 | 0.91 | 0.61 | 0.89 | 0.72 | 0.88 | 0.59 | 0.75 | 0.74 | 0.54 | 0.62 | 0.79 | 0.6 |
| 10.40 | 0.95 | 0.95 | 0.84 | 0.96 | 0.95 | 1.00 | 0.84 | 0.96 | 0.81 | 0.86 | 0.73 | 0.93 |
| 31.00 | 1.00 | 0.96 | 0.90 | 0.89 | 0.98 | 0.99 | 0.99 | 0.89 | 0.92 | 0.89 | 0.97 | 1.00 |

Figure 3 (Flushing out in fifth instar nymphs of *T. infestans* exposed to aerosolized DEET)

| Time (min) | control | | | DEET  (0,44) | | | DEET  (0,88) | | | DEET  (1,76) | | | IR3535  (1,76) | | | Tetramethrin  (0.006) | | |
| --- | --- | --- | --- | --- | --- | --- | --- | --- | --- | --- | --- | --- | --- | --- | --- | --- | --- | --- |
|  | R1 | R2 | R3 | R1 | R2 | R3 | R1 | R2 | R3 | R1 | R2 | R3 | R1 | R2 | R3 | R1 | R2 | R3 |
| 0 | 0 | 0 | 0 | 0 | 0 | 0 | 1 | 0 | 0 | 0 | 0 | 0 | 0 | 0 | 0 | 0 | 0 | 0 |
| 5 | 0 | 0 | 0 | 0 | 0 | 1 | 1 | 4 | 1 | 3 | 2 | 2 | 0 | 0 | 0 | 2 | 5 | 8 |
| 10 | 0 | 0 | 0 | 0 | 1 | 1 | 1 | 4 | 1 | 3 | 3 | 3 | 0 | 0 | 0 | 6 | 7 | 8 |
| 15 | 0 | 0 | 0 | 0 | 1 | 1 | 1 | 4 | 1 | 3 | 3 | 3 | 0 | 0 | 0 | 8 | 7 | 8 |
| 20 | 0 | 0 | 0 | 0 | 1 | 1 | 1 | 4 | 1 | 3 | 3 | 3 | 0 | 0 | 0 | 8 | 7 | 8 |
| 25 | 0 | 0 | 0 | 0 | 1 | 1 | 1 | 4 | 1 | 3 | 3 | 3 | 0 | 0 | 0 | 8 | 7 | 8 |
| 30 | 0 | 0 | 0 | 0 | 1 | 1 | 1 | 4 | 1 | 3 | 3 | 3 | 0 | 0 | 0 | 8 | 7 | 8 |
| 35 | 0 | 0 | 0 | 0 | 1 | 1 | 1 | 4 | 1 | 3 | 3 | 3 | 0 | 0 | 0 | 8 | 7 | 8 |
| 40 | 0 | 0 | 0 | 0 | 1 | 1 | 1 | 4 | 1 | 3 | 3 | 3 | 0 | 0 | 0 | 8 | 7 | 8 |
| 45 | 0 | 0 | 0 | 0 | 1 | 1 | 1 | 4 | 1 | 3 | 3 | 3 | 0 | 0 | 0 | 8 | 7 | 8 |
| 50 | 0 | 0 | 0 | 0 | 1 | 1 | 1 | 4 | 1 | 3 | 3 | 3 | 0 | 0 | 0 | 8 | 7 | 8 |
| 55 | 0 | 0 | 0 | 0 | 1 | 1 | 1 | 4 | 1 | 3 | 3 | 3 | 0 | 0 | 0 | 8 | 7 | 8 |
| 60 | 0 | 0 | 0 | 0 | 1 | 1 | 1 | 4 | 1 | 3 | 3 | 3 | 0 | 0 | 0 | 8 | 7 | 8 |
